# Concordance Rate between Clinicians and Watson for Oncology among Patients with Advanced Gastric Cancer: Early, Real-World Experience in Korea

**DOI:** 10.1155/2019/8072928

**Published:** 2019-02-03

**Authors:** Youn I Choi, Jun-won Chung, Kyoung Oh Kim, Kwang An Kwon, Yoon Jae Kim, Dong Kyun Park, Sung Min Ahn, So Hyun Park, Sun jin Sym, Dong Bok Shin, Young Saing Kim, Ki Hoon Sung, Jeong-Heum Baek, Uhn Lee

**Affiliations:** ^1^Department of Gastroenterology, Gil Medical Center, Gachon University, Incheon, Republic of Korea; ^2^Department of Oncology, Gil Medical Center, Gachon University, Incheon, Republic of Korea; ^3^Department of Radiation Oncology, Gachon University Gil Medical Center, Republic of Korea; ^4^Department of Surgery, Gil Medical Center, Gachon University, Incheon, Republic of Korea; ^5^Department of Neurosurgery and Director of AI-Based Precision Medicine, Gil Medical Center, Gachon University, Incheon, Republic of Korea

## Abstract

**Backgrounds/Aims:**

Watson for Oncology (WFO) is a cognitive technology that processes medical information by analyzing the latest evidence and guidelines. However, studies of the concordance rate between WFO and clinicians for advanced gastric cancer (AGC) are lacking.

**Methods:**

We retrospectively reviewed 65 patients with AGC who consulted WFO and the Gachon Gil Medical Center multidisciplinary team (GMDT) in 2016 and 2017. The recommendations of WFO were compared with the opinions of the GMDT. WFO provided three treatment options: recommended (first treatment option), for consideration (second treatment option), and not recommended.

**Results:**

In total, 65 patients (mean age 61.0 years; 44 males and 21 females) were included in the study. The concordance rate between WFO and the GMDT was 41.5% (27/65) at the recommended level and 87.7% (57/65) at the for consideration level. The main causes of discordance between WFO and the GMDT were as follows. First, WFO did not consider the medical history. Second, WFO recommended the use of agents that are considered outdated in Korea. Third, some patients wanted to be involved in a clinical trial. Fourth, some patients refused to use the biologic agents recommended by WFO for financial reasons as they were not covered by medical insurance.

**Conclusions:**

The concordance rate at the recommended level was relatively low but was higher at the for consideration level. Discordances arose mainly from the different medical circumstances at the Gachon Gil Medical Center (GMC) and the Memorial Sloan Kettering Cancer Center (MSKCC), the main WFO consulting center. The utility of WFO as a tool for supporting clinical decision making could be further improved by incorporating regional guidelines.

## 1. Introduction

Clinicians who treat patients with advanced gastric cancer (AGC) are challenged to personalize care using the rapidly expanding knowledge base [1]. Cancer-related databases include not only treatment guidelines but also, for example, drug approvals and up-to-date scientific evidence [1]. Management of this information is a challenge in personalized cancer management, as there is a little time for tracking and accessing relevant information [2].

Artificial intelligence systems have the potential to support clinicians in diagnosis, treatment, and predicting the prognosis of a variety of diseases [3]. Three clinical decision support systems (CDSS)—Clinical Oncology's Cancer Linq, Oncodoc, and International Business Machines (IBM)'s Watson for Oncology (WFO) [1, 2, 4]—have been used in medical oncology. Unlike other CDSSs, WFO recommends treatment options based on the literature, protocols, and the patient's chart and by learning from prior cases and the experiences of experts at the Memorial Sloan Kettering Cancer Center (MSKCC) [5].

During validation, WFO yielded a high concordance rate and multidisciplinary team approaches in medical oncology, including for breast [4–6], colon [7, 8], lung [7], and cervical [9] cancer. According to report conducted at MSKCC in the United States (US), the WFO-physician concordance rate was > 90% in 103 patients with nonmetastatic breast cancer [4–6]. In Thailand, among 211 cases the overall concordance rate was 83%; 89% for colorectal, 91% for lung, 76% for breast, and 78% for gastric cancer. Among 638 patients with breast cancer treated at Manipal Hospitals in Bangalore, India, a 90% concordance rate was observed between the recommendations of a multidisciplinary team (MDT) and WFO [9]. Among patients with cervical cancer [9], treatment recommendations were concordant in 299 (80.8%) of 370 patients: recommended for 277 and for consideration for 22.

WFO has not been validated in terms of the treatment concordance rate compared with multidisciplinary team approaches for patients with AGC, particularly in countries with a high incidence of AGC (e.g., Korea and Japan) [5, 10]. In Korea, the annual incidence of AGS is estimated to be 29,207; this represents > 4% of the global annual incidence [11–13]. Moreover, gastric cancer patients in Asian countries have a significantly higher 5-year survival rate than those in Western countries [14].

Therefore, we assessed the level of concordance between WFO and a GMDT for AGC treatment options and evaluated the causes of any discordance as an early, real-world experience in Korea.

## 2. Method

### 2.1. Study Design and Population

We compared the level of agreement between WFO (ver. 16.9; IBM Watson Health, Cambridge, MA) and the Gachon Gil Medical Center multidisciplinary team (GMDT) in terms of the treatment options recommended to 65 patients with AGC at the Gachon Gil Medical Center (GMC) in Inchon, Korea. All of the patients had been diagnosed with AGC and were either naïve to systemic therapy or had experienced disease recurrence after systemic and/or surgical treatment. All patients who presented with AGC within 1 month preceding acquisition of WFO (2016–2017) were included. Patients were excluded from this study if they had an Eastern Cooperative Oncology Group (ECOG) performance status of > 2, as further treatment is not considered for such patients. Patients with disease progression following systemic therapy (second line and beyond) were also excluded. The study protocol was reviewed and approved by the institutional review board prior to study initiation (IRB. GBIRB2017-292).

### 2.2. Watson for Oncology

The WFO treatment recommendations (which generally include several options) are categorized into the following three groups: recommended treatments, with a strong base of evidence; for consideration, which are suitable alternatives based on clinical judgment; and not recommended, which have specific contraindications or strong evidence against their use. Evidence supporting the recommended treatments is provided, as are any available case specific clinical trials, prescribing information, potential adverse reactions, and a comparison of treatment options.

After the WFO showed the treatment option for each patient as aforementioned categories, MDCT decided the final decision as reference to WFO's choice.

### 2.3. Data Collection and Concordance Determination

Patient data were abstracted from the medical records and entered manually into WFO by one trained oncology fellow. The GMDT had previously reviewed and recommended treatment regimens for all cases in 2016 and 2017; WFO analyzed the same cases.

If the GMDT recommendation for a case corresponded to the recommended or for consideration categories of WFO, it was defined as concordant. If the GMDT recommendation for a case was not available in WFO, it was designated discordant, which, together with the not recommended category, comprised the nonconcordant cases.

### 2.4. Data Analysis and Statistics

Descriptive statistics of the AGC cases were calculated using SPSS (ver. 20) and are presented as means ± standard deviation or medians (minimum; maximum). Concordance was expressed as percentage agreement. Categorical variables were analyzed by two-sided Pearson chi-squared test, and* p*-values of less than 0.05 were considered indicative of statistical significance. The cancer characteristics analyzed included patient age, cancer stage, and ECOG status. To control for these three parameters simultaneously, a logistic regression model was performed and odds ratios and 95% confidence intervals were reported.

## 3. Result

In total, 65 patients were assessed. The mean age of the patients was 61 years and most were males (n = 44, 67.7%). Of the 65 eligible patients, 90.7% had an ECOG performance status of 0 or 1. Among the AGCs, 38.5% (n = 24) and 12.3% (n = 8) were metastatic and recurrent, respectively (Table 1).

**Figure 1:**
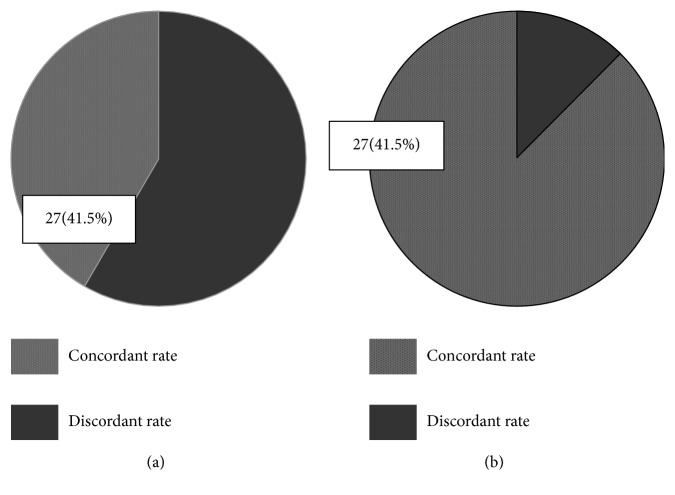
Concordance rate between WFO and the GMDT at (a) the recommended level and (b) the for consideration level. Treatment concordance between WFO and GMDT; (a) concordance of level of “recommended”; (a) concordant rate between WFO and GMDT (recommended); (b) concordant rate between WFO and GMDT (for consideration).

The percentage concordance between WFO and the GMDT at the recommended and for consideration levels was 41.5% (27/65) and 87.7% (57/65), respectively (Figure 1).

Regarding clinical factors, only the cancer stage (*p* <0.01) differed significantly between the concordant and discordant groups (Table 2).

Table 3 lists the results of a multivariate regression analysis of concordance as a function of patient age, Ro resection status, cancer stage, and performance status. Compared with stage II or III, treatment recommendations for AGC stage IV or recurrent disease were significantly (*p* = 0.02) more likely to be concordant.

Discordances between WFO and the GMDT were due to complex medical history of patients, clinicians' preferred chemotherapies, patient enrollment in clinical trials, and financial factors associated with the Korean National Health Insurance System (KNHIS) (Table 4).

## 4. Discussion

This retrospective observational study evaluated the concordance rate between WFO and the GMDT regarding treatment recommendations for AGC patients. The concordance rate was high at the for consideration level (87.7% [57/65]), but lower at the recommended level (41.5% [27/65]).

This is the first comparative study of AGC treatment recommendations by WFO and a MDT. The overall concordance rate in GC was 78% in Thailand, and 21.8% in China [5, 15, 16]. However, because these studies were published only as abstracts, detailed information on the number of AGC patients enrolled, disease stage, treatment options, and reasons for discordances was not available. In the present study, WFO was validated in an institution with a large number of AGC specialists. In Korea, the incidence and 5-year survival rate of GC are higher than those in Western countries. Indeed, the estimated annual incidence of GC in Korea, 29,207, represents > 4% of the global annual GC incidence [11–13], and the 5-year survival rate of GC is significantly higher in Korea than in Western countries [14].

WFO considers not only disease stage, postoperative pathologic findings, human epidermal growth factor receptor 2 (HER2) status, and general condition (critical disease scenarios and performance status), but also the age at diagnosis, sex, weight, histologic type, and prior therapy; these are not included in the National Comprehensive Cancer Network (NCCN) guidelines [15–17]. WFO facilitates AGC treatment decision making, especially in centers with a low incidence of GC and few or no specialists in AGC [5, 15, 16]. WFO also has the advantage of being constantly updated as new evidence emerges.

The low concordance rate between WFO and the GMDT among patients with AGC may be explained as follows: (1) some WFO-recommended chemotherapy regimens were not covered by the KNHIS, so the GMDT did not recommend such drugs, (2) a regimen known as S-1 (tegafur, gimeracil, and oteracil) plus cisplatin is routinely used in Korea but not in the US, (3) perioperative chemotherapy is generally used in Korea, but adjuvant chemoradiotherapy is used in the US, and (4) patients want to be enrolled in clinical trials.

Of note, stage was the main factor contributing discordance between WFO and GMDT treatment options in univariate and multivariate analysis (odds ratio 1.6,* p* = 0.02). Patients with stage IV or recurrent disease may be more likely to have a complex medical history, wish to be involved in clinical trials, and require chemotherapeutic agents not covered by the KNHIS (Table 4).

Table 4 lists the discordant factors between WFO and the GMDT. First, some WFO-recommended palliative chemotherapy regimens were not covered by the KNHIS. For example, a 72-year-old male patient with a history of cardiovascular disease had recurrent GC- and WFO-recommended paclitaxel, irinotecan, or docetaxel with carboplatin; these are not covered by the KNHIS. Also, WFO recommended trastuzumab with FOLFOX, XELOX, or mDCF to HER2+ patients with metastatic GC; these regimens are not covered by the KNHIS. In HER2+ patients with metastatic GC and grade 2 neuropathy, WFO recommended trastuzumab with S-1, irinotecan, or irinotecan/carboplatin, but these are not covered by the KNHIS. For patients with metastatic or recurrent GC, KNHIS does not cover paclitaxel/carboplatin, docetaxel/carboplatin, ramucirumab/irinotecan, irinotecan/carboplatin, or docetaxel/irinotecan as second-line chemotherapy regimens.

Second, a regimen known as S-1 (tegafur, gimeracil, and oteracil) plus cisplatin, is routinely used in Korea but not in the US. In Korea, patients with metastatic or recurrent gastric cancer usually receive S-1/capecitabine. The GMDT recommended the S-1 regimen, which is unknown to WFO, for patients with recurrent gastric cancer who had risk factors (elderly, history of chronic kidney disease, and ECOG 2).

Third, adjuvant perioperative chemoradiotherapy is not used in Korea; chemotherapy is generally recommended instead. In Korea, S-1 or XELOX are allowed as adjuvant regimens for patients with stage II or III disease.

WFO has several limitations. First, WFO does not reflect the specific loco-regional and socioeconomic circumstances [5, 16], e.g., coverage by the KNHIS. WFO recommendations are generally based on the experiences of the panel of cancer experts at MSKCC, supported by the medical literature. However, the experiences of physicians and the life circumstances, history, and treatment experiences of patients at MSKCC are not generally representative [18]. GC patients in Korea typically have different clinical characteristics from those at MSKCC [18]. Second, the survival benefit of WFO recommendations in AGC patients has not been validated [5, 19].

This study had several limitations. WFO was externally validated in a single Korean institute. However, the GMC was the first institute in Korea to introduce WFO. The GMC is a tertiary referral hospital that treats around 8% of the GC patients in Korea, and so this study has value. The utility of WFO should be validated in other patient groups.

In conclusion, WFO treatment recommendations were concordant with those of the GMDT in the majority of AGC patients. Most discordances were caused by differences in practice between the US, where WFO was trained, and Korea, where the GMDT was located. Therefore, region-specific customization of WFO would enable its use worldwide. Also, addition of local clinical factors would increase the level of sophistication of WFO as a CDSS.

## Figures and Tables

**Table 1:** Baseline characteristics of the patients with advanced gastric cancer (n = 65).

**Characteristic**	**n (**%**)**
Age, years	61.0 ± 11.7, 60.0 (52.0–71.0)
Gender (M: F)	44: 21
BMI	21.4 ± 3.5, 21.3 (18.6–23.8)
Chemotherapy	
First	51 (78.5%)
Second	10 (15.4%)
Third	4 (6.2%)
Adjuvant chemotherapy	23 (47.9)
Palliative chemotherapy	25 (52.1)
R resection	
R0	41 (63.1)
R1	2 (3.1)
R2	2 (3.1)
HER2+	9 (13.8)
Prior therapy	
Surgery	52 (80.0)
Chemotherapy	12 (18.5)
Surgery and chemotherapy	1 (1.5)
Stage	
IIA	9 (13.8)
IIB	12 (18.5)
IIIA	5 (7.7)
IIIB	5 (7.7)
IIIC	9 (13.8)
IV	25 (38.5)
Death	6 (9.2)
Performance	
ECOG 0	14 (21.5)
ECOG 1	45 (69.2)
ECOG 2	6 (9.2)

BMI, body mass index; HER2/neu, human epidermal growth factor receptor; ECOG,Eastern Cooperative Oncology Group.

**Table 2:** Univariate analysis of the concordance rate between WFO and the GMDT.

	Concordance (n = 27)	Discordance (n = 38)	*p*
**Age**	59.1 ± 12.3	62.0 ± 11.2	0.49
**Sex**			0.52
Male	17 (63.0%)	27 (71.1%)	
Female	10 (37.0%)	11 (28.9%)	
**BMI**	20.9 ± 3.6	21.6 ± 3.8	0.53
**Chemotherapy**			0.89
Adjuvant chemotherapy	14(51.9%)	19(50.0%)	
Palliative chemotherapy	13(48.1%)	19(50.0%)	
**R resection**			0.13
R0	19 (70.4%)	22 (57.9%)	
R1	1 (3.7%)	1 (2.6%)	
R2	2 (7.4%)	0 (0.0%)	
**HER2+**	4	5	0.83
**Stage**			< 0.01
II	6 (22.2%)	15 (39.5%)	
III	15 (55.6%)	4 (10.5%)	
IV or recurrent	6 (22.2%)	19 (50.0%)	
**Performance**			0.71
ECOG 0, 1	25 (92.6%)	34 (89.5%)	
ECOG 2, 3	2 (7.4%)	4 (10.5%)	

BMI, body mass index; HER2/neu, human epidermal growth factor receptor; ECOG,Eastern Cooperative Oncology Group.

**Table 3:** Multivariate analysis of discordance between WFO and the GMDT.

	Odds ratio (95% CI)	*p*
Age		0.59
< 60 years (reference)		
≥ 60 years	1.5 (0.2–9.3)	
R0 resection	0.3 (0.03–2.6)	0.28
Stage		0.02
II or III(reference)		
IV or recurrent	1.6 (0.3–9.4)	
Performance		0.81
ECOG 0, 1 (reference)		
ECOG 2, 3	1.5 (0.09–21.6)	

CI, confidence interval; WFO, Watson for Oncology; GMDT, Gachon Gil Medical Multidisciplinary Team; ECOG,Eastern Cooperative Oncology Group.

**Table 4:** Reasons for discordance (n = 38).

	n = 38	Stage IV or recurrent vs. others
(I) WFO does not consider patients' complex medical history		

Case 1. Recurrent gastric cancer with lymph node metastasis and a prior good response to FOLFOX (i) WFO recommended ramucirumab + Paclitaxel, (ii) GMDT recommended FOLFOX	1	1:0

Case 2. Recurrent gastric cancer with solitary bone metastasis (i) WFO recommended ramucirumab + paclitaxel, (ii) GMDT recommended palliative radiation therapy	1	1:0

(II) Adjuvant therapy in patients who underwent curative resection of gastric cancer who were younger and in good condition (i) WFO recommended postoperative adjuvant fluoropyrimidine- based chemoradiation to patients with stage II or III disease, (ii) GMDT recommended S-1 monotherapy	4	0:4

(III) Adjuvant therapy in patients who underwent curative resection of gastric cancer who were older or had a complex medical history (i) WFO recommended postoperative adjuvant fluoropyrimidine-based chemoradiation to patients with stage II or III disease, (ii) GMDT recommended S-1 monotherapy	9	0:9

(IV) Adjuvant therapy in patients with locally advanced gastric cancer who underwent curative resection of gastric cancer (i) WFO recommended Capecitabine+Oxaliplatin (ii) GMDT recommended S-1 monotherapy	6	0:6

(V) Metastatic gastric cancer with HER2/neu (i) WFO recommended dose modified DCF or FOLFOX (ii) GMDT recommended capecitabine+cisplatin or 5-FU+cisplatin	11	11:0

(VI) Patients wanted to be involved in a clinical trial	3	3:0

(VII) Financial problem, WFO recommended a biologic agent, but patient refused for financial reasons (not covered by the KNHIS)	3	3:0

FOLFOX, Oxaliplatin, Folinic Acid, and 5-Fluorouracil; 5-FU, 5 Fluorouracil; KNHIS, Korean National Health Insurance System; HER2/neu, human epidermal growth factor receptor; DCF, docetaxel, cisplatin, and 5FU.

## Data Availability

The data used to support the findings of this study are available from the corresponding author upon request.
